# Estimating the effect on obesity of delaying tax-based interventions in Mexico: A modeling study

**DOI:** 10.1371/journal.pmed.1004769

**Published:** 2025-10-23

**Authors:** Martha Carnalla, Francisco Reyes-Sánchez, Alexis Alonso-Bastida, Alan Reyes-García, Alessio Hernández-Rojas, C. Gabriela García, Isabel Junquera-Badilla, Ana Basto-Abreu, Boyd Swimburn, Juan A. Rivera, Tonatiuh Barrientos-Gutiérrez

**Affiliations:** 1 Centro de Investigación en Salud Poblacional, Instituto Nacional de Salud Pública, Cuernavaca, México; 2 School of Population Health, University of Auckland, Auckland, New Zealand; Waymark, UNITED STATES OF AMERICA

## Abstract

**Background:**

The World Health Organization (WHO) launched the Acceleration Plan to STOP Obesity, highlighting the urgent need for timely implementation of proven interventions. Fiscal policies, including taxes on sugar-sweetened beverages (SSB) and non-essential energy-dense foods (NEDF), are among the most cost-effective strategies to reduce obesity rates. Delays in adopting or strengthening these measures can undermine their impact, and the consequences of postponing such policies remain unmeasured. We aimed to estimate the expected impact of delaying doubling the SSB and NEDF tax in Mexico.

**Methods and findings:**

We simulated a closed cohort of Mexican adults aged 20 years and over from 2021 to 2040. The simulated sample corresponded to the combination of the 2020−22 Health and Nutrition Surveys, which contained anthropometric and demographic information representative at a national level. We projected annual average Body Mass Index (BMI), obesity prevalence, deaths averted, and years lived without obesity (YLWO) under four scenarios: status quo and doubling the current tax on SSB and NEDF in 2025, 2030, and 2035. BMI was projected from 2021 to 2040 using Hall’s microsimulation weight change model, and a Mexican projection of total energy intake. To simulate deaths, we estimated the probability of all-cause mortality by BMI category from the National Population Council projections of the Mexican population by age and year. By 2040, doubling the taxes in 2025 resulted in an obesity prevalence of 41.6% (95% Uncertainty Interval [40.2,43.1]) in contrast to the status quo scenario (44.5%, 95% Uncertainty Interval [43.2,45.8]), and 170,600 deaths averted (95% Uncertainty Interval [130,900, 210,200]) and 25,031,900 (95% Uncertainty Interval [19,048,500, 31,015,300]) YLWO gained. A delayed intervention in 2035 resulted in an obesity prevalence of 41.7% (95% Uncertainty Interval [40.4,43.1]), 38,900 deaths averted (95% Uncertainty Interval [29,600, 48,200]), and 4,473,700 (95% Uncertainty Interval [3,378,900, 5,568,500]) YLWO gained. Our results apply only to individuals aged 20 years or older in 2021, excluding cohorts reaching age 20 between 2022 and 2040.

**Conclusions:**

Our results emphasize the urgency of advancing WHO’s Acceleration Plan to STOP Obesity. Postponing evidence-based interventions is estimated to exacerbate the burden of obesity, mortality, and suffering.

## Author summary

**Why was this study done?:**

- Structural interventions to control obesity, such as unhealthy food and beverage taxes, are available and cost-effective. Yet, they are subject to significant delays in approval and implementation.
- Mexico led the implementation of taxes on sugar-sweetened beverages (SSB) and non-essential energy-dense foods (NEDF). Still, despite recommendations to double taxes since 2018, no action has been taken.
- Delays in the implementation of structural interventions to control obesity have important impacts, but these are rarely discussed or estimated. Thus, we aimed to estimate the cost in terms of years lived without obesity and deaths of delaying doubling health taxes in Mexico.

**What did the researchers do and find?:**

- Using national data from Mexican adults, we modeled the impact of waiting to double existing taxes on SSB and NEDF. We simulated the Mexican adult population and compared what would happen if taxes were doubled in 2025, 2030, or 2035.
- Doubling the taxes in 2025 could prevent more than 170,000 deaths and add 25 million healthier life years by the year 2040, compared to waiting until 2035 to double the taxes.

**What do these findings mean?:**

- Delaying the implementation of effective policies, such as increasing taxes on unhealthy products and beverages, has serious consequences: the longer these measures are postponed, the more people will be at risk- and die- from obesity-related diseases.
- This study highlights the urgent need for policymakers to act quickly on evidence-based solutions to reduce obesity and its negative outcomes, and for scientists to consider the cost of delay when modeling potential health interventions.
- Our model has important limitations. We assume that consumption trends for NEDF and SSB will slow over time, which may not reflect future real-world changes. We also do not account for the younger generation’s consumption patterns, who may respond differently to taxes.

## Abstract 3

In a modelling study, Martha Carnalla and colleagues investigate the effects doubling the current tax on sugar-sweetened beverages and non-essential energy-dense foods would have on obesity levels, by simulating a closed cohort of Mexican adults.

## Introduction

The rising prevalence of obesity poses a significant threat to global health. Preventing further increases in obesity is critical to achieving the 2030 Sustainable Development Goal (SGD) of reducing premature mortality from non-communicable chronic diseases by 30% [1]. To facilitate policy decision-making and actions in countries across the world, in 2022, the World Health Organization (WHO) launched the Acceleration Plan to STOP Obesity [2]. The Plan proposes cost-effective and evidence-based actions such as fiscal policies, marketing restrictions, or food labeling, among others, to be rapidly implemented by countries.

A key aspect of the Acceleration Plan to STOP Obesity is time. The Plan urges countries to focus resources and capabilities to implement interventions promptly and according to their context. Interestingly, while modeling studies have estimated the cost-effectiveness of obesity policies [3,4], no study has assessed the impact of delaying policy decision-making. Climate change and vaccination studies generate estimates of the cost-of-delay [5,6], yet no similar efforts are being conducted for obesity. This is a key input for policy decision-making since quantifying the human cost of delay will provide a clear picture of the costs of waiting to implement recommended measures.

Mexico has been at the forefront of innovation for obesity-related policy interventions. In 2014, Mexico introduced a 10% tax on sugar-sweetened beverages (SSB) and an 8% tax on non-essential energy-dense foods (NEDF), being one of the first countries in the Americas to establish such policies [7]. In 2024, the WHO recommended the implementation of SSB taxes and suggested the implementation of unhealthy food taxes to all countries [8]. In Mexico, calls to double the tax on SSB and NEDF to further increase their impact have been made since 2018 [9]. Several initiatives have been presented in the Chamber of Representatives, yet to date, no increase in tax rates has been approved. Thus, we considered the delay in approving doubling the SSB and NEDF tax a valuable case for modeling the cost of delayed policy decision-making.

We aimed to estimate the human cost of delaying the approval of doubling SSB and NEDF taxes in Mexico. For that purpose, we compared the expected impact in 2040 of doubling the tax in three potential years: 2025, 2030, and 2035. The cost of delay was assessed by calculating obesity prevalence, years lived without obesity, and deaths averted compared to no increases in taxes.

## Methods

**Fig 1:**
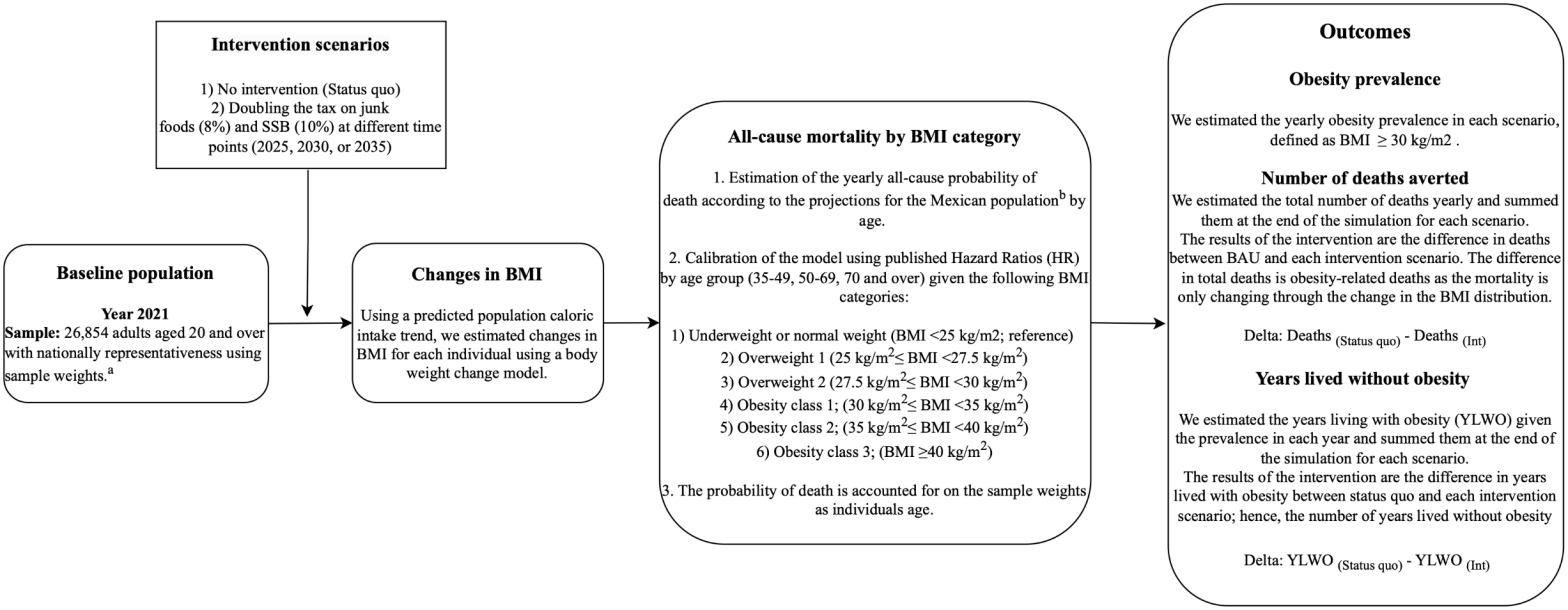
Step-by-step summarized modeling process. ^a^Sample weights expand to nationally represent the adult population. ^b^Estimated by the National Population Council of Mexico. BMI: body mass index. YLWO: years lived without obesity.

Fig 1 shows the structure of the model. Briefly, we used data from adults (20–100 years) from the National Health and Nutrition Survey (ENSANUT) 2020−22 to simulate the Mexican population from 2021 to 2040. To simulate body weight changes, we combined an estimated trend of energy intake and the dynamic weight change model proposed by Hall and colleagues [10]. We explored two scenarios: (1) status quo (i.e., historical trend of total energy intake (TEI) where the 10% and 8% tax on SSB and NEDF is maintained), and (2) doubling the tax on SSB and NEDF (20% and 16%) in 2025, 2030, or 2035. We estimated an annual all-cause mortality rate dependent on the individual’s body mass index (BMI) category and age. All scenarios were simulated up to the year 2040. More details of the simulation process are presented in the next subsections.

### Population

The baseline population was ENSANUTs 2020–2022 adult population. Each ENSANUT is a cross-sectional, multistage, stratified, and cluster-sample survey representative of national, regional, rural, and urban populations. The design and methods of each survey were described elsewhere [11]. Each survey research protocol was approved by the Ethics, Biosafety, and Research Committees of the National Institute of Public Health of Mexico, and informed consent was obtained from each participant. For our analysis, we assumed that the ENSANUT 2020–2022 represented the Mexican population of the year 2021 (the mid-point between 2020 and 2022). The wave-specific survey weights of the ENSANUT 2020–2022 were divided by the number of ENSANUTs (n = 3) and then calibrated with raking, a sampling balancing method to match the Mexican population reported by the National Population Council of Mexico (CONAPO) for 2021 (S1 Appendix, Section 2). We simulated adults (20–100 years) in 2021 (from the ENSANUT 2020–2021) for 2021–2040 as a closed cohort. Thus, by 2040, the simulated population consisted of adults aged 39–100 years. We did not consider new cohorts entering the model. To simulate the population changes over the study period, we considered projections of the Mexican population (mid-year population, years: 2021–2040) from CONAPO [12] (further details in section 5 in S1 Appendix).

### Anthropometric data

Body weight and height were measured following the same standardized procedures across the ENSANUT waves (2020–2022) [13]. We excluded individuals without weight or height data (n = 256), extreme values of height (<130 cm or >200 cm; n = 213), BMI (<10 or >59 kg/m^2^; n = 38), and pregnant or lactating women (n = 746). The analytical sample included a total of 26,854 adults.

### SSB and NEFD consumption

Dietary data was collected from a nationally representative subsample of the ENSANUTs 2020–2022 using a semi-quantitative Food Frequency Questionnaire (SFFQ; n = 4,091). The SFFQ was collected in person by trained interviewers, who asked about frequency, portion size, and number of servings consumed from 140 food and beverage items during the seven days before the interview [14]. Following tax law definitions, items were grouped into four main groups: taxed beverages, untaxed beverages, taxed foods, and untaxed foods. For this analysis, we only considered the intake of taxed items. The reported intake was converted into milliliters or grams per day and then to energy intake (kcal) using the food composition table compiled by the Mexican National Institute of Public Health.

### Intervention scenarios

We estimated annual outcomes under the following scenarios:

(1) Status quo. In this scenario, no intervention is considered so that the historical trend of total energy intake (observed data 2000–2021) continued undisturbed from 2021 to 2040. To project the total energy intake (TEI), our first approach was to consider a linear trend. This trend fitted well to the estimations of Mexican energy intake (adjusted R-square >0.96; section 3.1.2 in S1 Appendix); however, it could overestimate results due to the large simulation period (from 2021 to 2040). To prevent that situation, we used a linear fit for the first years from baseline (2021–2025) and then slope reductions in the subsequent years, assuming that energy intake would continue to increase, but at a slower pace, as susceptible people to the obesogenic environment are depleted. The slope reductions were taken from a cancer incidence model, called Nordpred [15,16]: a 25% reduction in 2026–2030, a 50% during 2031–2035, and a 75% in the subsequent years (Fig C in S1 Appendix). Nordpred is a mathematical model designed to improve long-term predictions, assuming the slope will eventually flatten. The projection of intake with the Nordpred reductions was previously compared with alternative projections: linear fit, root square fit, and a Gompertz model [17]. The uncertainty intervals of the Nordpred obesity overlapped with those of the alternative projections. In addition, the Nordpred-based results were consistent with projections of obesity from a multinomial model proposed by Ward and colleagues [18]. An overview of the Nordpred-based projection can be found in section 3.1 in S1 Appendix.
(2) Doubling the current tax on SSB and NEDF. Building on Mexico’s 2014 tax implementation (10% SSB, 8% NEDF) that reduced purchases by 7.6% (95% Confidence Interval: Not available) and 6.0% (95% Confidence Interval [3.8,8.3]), respectively [19,20], we modeled the effect of adding an extra 10% SSB and 8% NEDF tax to the existing fiscal measures starting at different time points. We assumed that these reductions (7.6% SSB, 6.0% NEDF) equaled the reduction in consumption reported in the SFFQ. For instance, a given individual in the SSFQ with SSB and NEDF intake of 100 kcal/day and 200 kcal/day, respectively, would reduce 19.6 kcal/d (= 100*0.076 + 200*0.06) of their total TEI by day. Since the SSFQ is a subsample (n = 4,091) of the baseline sample (n = 26,854), we could not estimate the reductions of intake per individual in the baseline. Thus, we estimated the overall average reduction of TEI from the SSFQ (%) and used this average for our simulations. The 7.6% and 6.0% reductions in SSB and NEDF consumption translated to an average reduction of 0.93% (95% Uncertainty Interval [0.72,1.15]) of the TEI. This reduction in TEI was applied to the historical trend, assuming that the total reduction would be reached two years after implementing the tax, giving time to fully comply with the tax (Fig F in S1 Appendix). No substitution effects were modeled, consistent with Mexican evidence showing no significant increase in untaxed food purchases [21] and limited cross-category substitution [19], with increases in bottled water with no calories. The expected caloric reductions were introduced in the model at the expected year of implementation: 2025, 2030, or 2035.

### Changes in body mass index

For each scenario, we ran Hall’s weight change model considering the corresponding energy intake projection to simulate BMI from 2022 to 2040. Briefly, Hall’s weight change model is a microsimulation model that estimates body weight for each individual as the sum of extracellular fluid, glycogen, fat mass, and lean tissue, dependent on sodium and energy intake, energy expenditure, and individual characteristics such as sex, age, and physical activity level [10]. Changes in BMI were estimated annually, assuming that the individual’s height remained constant over time. Then, we classified the individuals into one of the following categories of BMI: (1) underweight or normal weight (reference category, BMI < 25 kg/m^2^), (2) overweight 1 (25 kg/m^2^ ≤ BMI < 27.5 kg/m^2^), (3) overweight 2 (27.5 kg/m^2^ ≤ BMI < 30 kg/m^2^), (4) obesity class 1 (30 kg/m^2^ ≤ BMI < 35 kg/m^2^), (5) obesity class 2 (35 kg/m^2^ ≤ BMI < 40 kg/m^2^), or (6) obesity class 3 (40 kg/m^2^ ≤ BMI). We estimated the prevalence of each BMI category by year and age.

### All-cause mortality by BMI category

To simulate mortality, we estimated the probability of all-cause mortality (*P[D]*) from CONAPO’s projections of the Mexican population by age and year. The estimation process is exemplified for adults aged 95 in 2021 in Section 5 in S1 Appendix. We assumed that people aged 100 years had a 100% probability of dying (*P[D]* = 1); i.e., the maximum age considered was 100 years.

The probability of all-cause mortality *P[D]* was decomposed across the BMI categories considered using hazard ratios (HRs) of all-cause mortality (shown in Table 1) and the methodology followed by Allison et, al. [22] An overview of that methodology is presented in Section 5.1 in S1 Appendix.

**Table 1:** Magnitude, characteristics, and data source of the hazard ratios for death considered in the analyses.

Risk factor exposure	Hazard ratio per 5 units of BMI (95% CI)	Design	Source	Hazard ratio by BMI category*
Age 35–49 years	1.52 (1.47, 1.56)	Meta-analysis	The Global BMI Mortality Collaboration et, al. 2016	1) BMI < 25.0: HR = 1.00 2) 25.0 ≤ BMI < 27.5: HR = 1.23 3) 27.5 ≤ BMI < 30: HR = 1.52 4) 30.0 ≤ BMI < 35.0: HR = 2.31 5) 35.0 ≤ BMI < 40.0: HR = 3.51 6) 40.0 ≤ BMI: HR = 5.34
Age 50–69 years	1.37 (1.35, 1.39)			1) BMI < 25.0: HR = 1.00 2) 25.0 ≤ BMI < 27.5: HR = 1.17 3) 27.5 ≤ BMI < 30: HR = 1.37 4) 30.0 ≤ BMI < 35.0: HR = 1.88 5) 35.0 ≤ BMI < 40.0: HR = 2.57 6) 40.0 ≤ BMI: HR = 3.52
Age 70–89 years	1.21 (1.17, 1.25)			1) BMI < 25.0: HR = 1.00 2) 25.0 ≤ BMI < 27.5: HR = 1.10 3) 27.5 ≤ BMI < 30: HR = 1.21 4) 30.0 ≤ BMI < 35.0: HR = 1.46 5) 35.0 ≤ BMI < 40.0: HR = 1.77 6) 40.0 ≤ BMI: HR = 2.14

BMI: Body Mass Index. * Estimated using the “Hazard ratio per 5-units BMI (95% CI)” column, assuming an exponential function for the hazard ratios. Changes per 5 units of BMI are considered from 25 kg/m^2^. For example, for the “Age 35 to 49 years” group:

HR(BMI category 1) = exp{log(1.52) * (25-25)/5} = 1.0;

HR(BMI category 2) = exp{log(1.52) * (27.5-25)/5} = 1.23;

HR(BMI category 3) = exp{log(1.52) * (30-25)/5} = 1.52;

HR(BMI category 4) = exp{log(1.52) * (35-25)/5} = 2.31;

HR(BMI category 5) = exp{log(1.52) * (40-25)/5} = 3.51;

HR(BMI category 6) = exp{log(1.52) * (45-25)/5} = 5.34.

The HRs for all-cause mortality by BMI, shown in Table 1, were derived from a meta-analytic study by the Global BMI Mortality Collaboration and colleagues (2016) [23]. The HRs were reported for people aged 35–89 years. For people aged > 89, we assumed they had the same HR as the group of 70–89. For people aged <35, we just used the probability of all-cause mortality (*P[D]*); i.e., we did not estimate mortality by BMI.

We decomposed *P[D]* starting in the year 2026 of the simulation, considering that the HRs were estimated five years after follow-up to avoid bias and confounding. In summary, given an individual *j* in the BMI category *Ai* in the year *y*, we assigned a probability of dying during the year y (*PD(y)*) as:


$$PD_{j}\left(y\right)=\{\begin{array}{c} P[D|Ai,\;\;if\;age_{j}\;\geq \;\;35\;\;and\;y\geq 2026\\ P\left[D\right]\;\;\;\;\;\;\;\;\;\;\;\;\;\;\;\;\;if\;age_{j}\;<\;\;35\;\;or\;y\;<\;2026\;\;\\ 1\;\;\;\;\;\;\;\;\;\;\;\;\;\;\;\;\;\;\;\;\;\;\;\;\;\;\;\;\;\;\;\;\;\;\;\;\;\;\;if\;age_{j}\;=\;100\;\;\;\;\;\;\;\;\;\;\;\;\;\;\;\;\; \end{array}$$ (1)

where *P[D|A*_*i*_*]* is the probability of all-cause mortality in the BMI group *A*_*i*_. Both *P[D|Ai]* and *P[D]* depended on the year *y* and the age of the individual*.* The probabilities of death *PD(y)* were estimated for the status quo scenario using the projections of the Mexican population from CONAPO. The same values of *PD(y)* were used for the intervention scenarios. Thus, the difference in deaths between the status quo and the intervention scenarios was driven by the difference in the prevalence of the BMI categories.

### Outcomes

#### Obesity prevalence.

We estimated the annual BMI of the individuals in the sample, defined as weight in kg divided by height in meters squared. Individuals with a BMI ≥ 30 kg/m^2^ were assigned to the obesity category, according to WHO’s BMI classification.

#### Deaths averted.

We estimated the deaths averted as the difference in deaths between the status quo and the intervention scenarios. The estimation of annual deaths is presented in Section 6 in S1 Appendix.

#### Years lived without obesity.

We computed the years lived (adapted from Selvin, 2008) [24] and the years lived without obesity (YLWO) (adapted from Jagger, 2014) [25] using a cross-sectional approach by year (cross-sectional versions of the life-table life-years and disease-free life-years) as follows:


$$\text{YLWOs saved}=\sum_{y=\text{2}\text{0}\text{2}\text{1}}^{\text{2}\text{0}\text{4}\text{0}}\sum_{m=\text{2}\text{0}}^{\text{9}\text{5}+}\left({{YLWO}_{m,y}}_{INT}-{{YLWO}_{m,y}}_{Status\;quo}\right);$$

$${YLWO}_{m,y}=(\text{1}-\pi_{m,y}){YL}_{m,y}$$

$${YL}_{m,y}=\left(l_{m,y}-d_{m,y}\right)+a_{m,y}d_{m,y}$$

where ${YL}_{m,y}$ are the cross-sectional years lived in age group *m* and year *y* and ${YLWO}_{m,y}$, the cross-sectional years lived without obesity. The mid-year population is represented by $l_{m,y}$, $d_{m,y}$ are the deaths and $\pi_{m,y}$ the prevalence of obesity, all in each age group *m* and year *y*. The quantity ($l_{m,y}-d_{m,y}$) is the number of individuals who survived the entire interval (from age *x* to age *x* + 1); these individuals contribute $\left(l_{m,y}-d_{m,y}\right)$ to years lived. $a_{m,y}d_{m,y}$ is the contribution of years lived by the individuals who died in the interval (from age *x* to age *x* + 1). We assume an average survival time of one half-year (a_m,y_ = 0.5), so they contribute to $\text{0}.\text{5}\times d_{m,y}$ years lived.

All the assumptions and parameters included in the model are presented in section 9 and Table J in S1 Appendix.

### Uncertainty intervals

We considered 1,000 replicated survey weights using bootstrap [26] to account for the uncertainty from the sample. For each set of replicated survey weights, we used the Monte Carlo approach to simulate the tax effect, and the meta-analytic HRs (Table J in S1 Appendix summarizes the simulated parameters). Then, we re-estimated the outcomes for each simulation. We took the 2.5 and the 97.5 percentiles of the outcome samples as the uncertainty intervals. More details about the uncertainty intervals are presented in section 7 in S1 Appendix. Hence, we are considering the uncertainty of the sample, the trend in energy intake change, the tax effect, and the meta-analytic mortality HRs. We did not incorporate uncertainty from the Hall’s model parameters (Table C in S1 Appendix) because it is a deterministic model, and we lacked information about the functional form of their covariance. Accounting for this uncertainty could lead to an over- or underestimation of the uncertainty intervals.

### Sensitivity analysis

As a sensitivity analysis, we considered a linear projection of the energy intake as an alternative trend to the Nordpred-based projection to consider a less conservative scenario.

This study is reported as per the Transparent Reporting of a Multivariable Prediction Model for Individual Prognosis Or Diagnosis (TRIPOD) statement (S1 TRIPOD Checklist). The code used in the analysis is available in Zenodo [https://doi.org/10.5281/zenodo.14901792].

## Results

**Fig 2:**
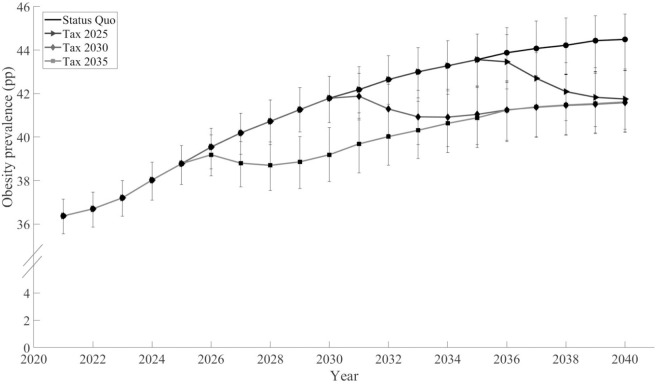
Predicted obesity prevalence from 2021 to 2,040 in the status quo and doubling the tax on sugar-sweetened beverages and non-essential energy-dense foods in the years 2025, 2030, and 2,035 in a closed cohort of 20-year-olds and over.

Fig 2 presents the obesity prevalence from 2021 to 2040 for the status quo and each intervention scenario. Doubling the tax on SBB and NEDF in 2025 resulted in an obesity prevalence of 41.6% (95% Uncertainty Interval [40.2,43.1]) in 2040, in contrast to the status quo, where the prevalence was 44.5% (95% Uncertainty Interval [43.2,45.8]). Doubling the tax on SBB and NEDF in 2030 would result in 41.6% (95% Uncertainty Interval [40.2,43.0]) obesity prevalence, and doubling the tax in 2035 resulted in 41.7% (95% Uncertainty Interval [40.4,43.1]) obesity prevalence.

Table 2 shows the predicted cumulative deaths averted and YLWO gained from 2021 to 2040 for each intervention scenario compared to the status quo, showing that implementing the tax earlier yields the greatest benefits. By doubling the tax in 2025, 170,600 deaths would be averted (95% Uncertainty Interval [130,900, 210,200]); in 2030, 110,400 (95% Uncertainty Interval [84,000, 136,800]); and in 2035, 38,900 (95% Uncertainty Interval [29,600, 48,200]). Similarly, doubling the tax in 2025 would result in 25,031,900 YLWO (95% Uncertainty Interval [19,048,500, 31,015,300]); doubling the tax in 2030, 14,356,500 YLWO (95% Uncertainty Interval [10,916,800, 17,796,200]); and doubling the tax in 2035, 4,473,700 YLWO (95% Uncertainty Interval [3,378,900, 5,568,500]). So, implementing the tax in 2025 would avert 54.5% more deaths and would gain 74.4% more YLWO compared to 2030, and 338.6% more deaths averted, and 459.5% more YLWO gained compared to 2035.

**Table 2:** Predicted cumulative deaths averted and years lived without obesity gained from 2021 to 2040 for doubling the SSB and NEDF tax in the years 2025, 2030, and 2,035 in Mexican adults aged 20 and over in 2021.

Intervention scenario	Deaths averted (thousands)	YLWO saved (thousands)
	n (p2.5, p97.5)	n (p2.5 – p97.5)
Doubling tax in 2025	170.6 (130.9, 210.2)	25,031.9 (19,048.5, 31,015.3)
Doubling tax in 2030	110.4 (84.0, 136.8)	14,356.5 (10,916.8, 17,796.2)
Doubling tax in 2035	38.9 (29.6, 48.2)	4,473.7 (3,378.9, 5,568.5)

YLWO: Years Live Without Obesity, SSB: sugar-sweetened beverages, NEDF: non-essential energy-dense foods.

## Discussion

We aimed to model the human costs associated with delaying the decision to double the SSB and NEDF tax in Mexico. We found that the same intervention, when implemented in 2025, 2030, or 2035 yielded similar results in obesity prevalence by 2040 (∼41.6%), a reflection of the relative impact of the intervention. Despite achieving the same prevalence, implementing taxes earlier resulted in significantly healthier lives. Implementing the intervention in 2025 would lead to 74% more years lived without obesity and 54% more deaths averted compared to intervening in 2030 and to 459% and 339% compared to intervening in 2035. These results highlight the human costs of waiting to implement a widely recommended, evidence-based intervention, such as SSB and NEDF taxes.

Quantifying the cost-of-delay of a public health intervention is rare. While implementing evidence-based interventions as soon as possible is common sense, policy change is complex, and decisions may be delayed for various reasons. Studies quantifying the cost-of-delay have appeared in climate change and vaccination areas [5,6]. Quantifying the cost of delay may be more frequent in these areas due to their perceived urgency. Yet, for many countries, implementing population-level interventions to reduce cardiometabolic disease is just as urgent. Mexico declared an epidemiological emergency due to obesity and diabetes in 2016 [27]. Calls to increase the SSB tax in Mexico have been made at least since 2018, yet up to February 2025, no change has occurred [9]. By estimating the human cost of delay, we aim to quantify the burden of disease associated with postponing decision-making for evidence-based and widely accepted policies, such as the human cost borne by the Mexican population due to delays in approving the increase of the SSB and NEDF taxes.

Modeling studies on unhealthy food and beverage taxes have mainly focused on estimating the expected impact of different scenarios over a certain timeframe. Our analysis adds a new layer of information by considering the cumulative gains of intervening sooner rather than later. This is best explained by our results when looking at the prevalence of obesity at the end of the simulation period. By 2040, all intervention scenarios converge to a similar prevalence (∼41.6%), which could be interpreted as the three scenarios providing the same benefit. However, early interventions result in larger cumulative benefits. The number of deaths averted is twice as high when the intervention is implemented in 2025 than in 2035. While this conclusion could be self-evident, its quantification is not. Our study shows that the population benefits of an intervention do not depend exclusively on its theoretical effectiveness but also on how quickly it is implemented.

By modeling the same intervention at different time points, we shed light into the controversy of expected versus observed obesity impacts after population interventions. Typically, the expectation is that obesity prevalence will decrease relative to pre-intervention levels (e.g., an intervention is considered effective if it decreases the obesity prevalence from 20% to 18%). However, the impact of an intervention is influenced by the energy intake trend prior to implementation. This is illustrated in our model, which captures the positive historical trend in energy intake and assumes this trend will stabilize over time (Norpred-based specification; Fig F in S1 Appendix). Thus, our simulation begins with a steep positive trend in energy intake that gradually diminishes, approaching zero by the end of the simulation period. When comparing the impact of each scenario five years after implementation with pre-intervention prevalence, intervening in 2035 appears to be the most effective. In this scenario, obesity prevalence decreases from 43.6% in 2035 to 41.7% in 2040, whereas in the 2025 scenario, prevalence increases from 38.8% in 2025 to 39.2% in 2030 (Table L in S1 Appendix). This difference arises because in 2025, the TEI trend is steeper, requiring a stronger intervention to achieve the same pre- to post-effect. When estimating the effects of population weight interventions, it is preferable to compare the expected results with a counterfactual projection that incorporates historical energy trends (note that all scenarios yield the same effect compared to the status quo). If counterfactual projections are unavailable, researchers should always consider historical energy intake trends to accurately assess pre- to post-intervention results.

Our findings are consistent with modeling studies from other middle-income countries. A 20% SSB tax was projected to reduce adult obesity by 3.8 percentage points (pp) in Thailand over 10 years [28], by 6.7 pp in Brazil over a 10-year period [29], and by 3.0 pp in India, where it was also estimated to reduce diabetes incidence by 1.6 pp over the same timeframe [30]. These results, along with our projections for Mexico, support the effectiveness and feasibility of fiscal policies targeting SSB and NEDF to reduce obesity and related chronic diseases in diverse economic and social contexts.

Our study has some limitations. First, a standard limitation of this type of simulation analysis is its strong dependence on input assumptions, mainly the meta-analytic mortality HR, which could substantially influence the estimated outcomes. However, other inputs derived from the Mexican context, such as the energy intake trend and the tax effect, which derived from studies conducted in the context of the Mexican national policy environment. Second, we are decreasing a fixed percentage caloric reduction to the historical TEI trend when doubling the tax. We assumed that the caloric trend maintains the same proportion of energy intake from SSB and NEDF over time. If the intervention were to reduce the proportion of TEI contributed by SSB and NEDF, we could overestimate the results; still, that will not affect our conclusion of larger benefits when implementing the intervention sooner. Third, the future status quo is unclear, and we are assuming a positive energy intake trend, with a gradually decreasing slope over time (Nordpred-based specification), reflecting the idea that energy consumption in the population will eventually plateau, as it cannot increase indefinitely. This assumption has an important effect on the magnitude of the impact on BMI and obesity. While we consider this approach conservative, it may underestimate the potential increase in TEI over time, as seen in section 8 in S1 Appendix where we substituted the Nordpred with a Linear trend. However, the uncertainty considered in the TEI trend captures a wide range of values that overlap in both assumptions (Norpred and linear trend) as shown in Fig C in S1 Appendix. Fourth, our modeled consumption changes imply price elasticities of −0.76 (7.6% reduction divided by 10% price increase) in SSB and −0.75 (6% reduction divided by 8% price increase), in NEDF. These estimates are more conservative than price elasticity studies reported by Mexican evidence on household-level SSB elasticity estimates (−1.16) [31] and international evidence on SSB (−1.59) [32] and on salty and snack chips (−1.95) [33]. Fifth, we are simulating a closed cohort (i.e., new generations do not enter), which might underestimate obesity prevalence given the positive TEI trends observed in children and adolescents. The main reason is that the TEI intake for younger generations is likely to have a different trajectory, given the latest interventions implemented in Mexico—which are expected to have a greater impact on children and adolescents—and we have yet to analyze the results in these generations. Hence, the uncertainty of their status quo is high. Our results in year 2040 apply only to the generations of adults aged 20 and over in 2021; that is, it does not apply to new generations of 20-year-olds from 2022 to 2040. Sixth, we are using a meta-analytic HRs for obesity-related mortality. The constant hazard assumption is dependent on the time of follow-up [34]. We are modeling 19 years, and the median follow-up of the studies included in the meta-analysis is 13.7 years. However, we are considering simulation upon the same age groups as the meta-analysis: 35–49 (14 years range), 50–69 (19 years range), and 70–89 (19 years range), so our extrapolation is limited to five years for groups 50–69 and 70–89. The potential bias is reduced since we are considering the change in the HR as individuals move across age groups.

Our results highlight the need to accelerate action to reduce obesity, as championed by the WHO. Delays in the implementation of cost-effective, evidence-based interventions increase the burden of disease, death, and suffering in the population. The longer individuals live with obesity, the higher their risk of developing serious complications. Our analysis shows how delays in implementing interventions can significantly harm population health. Additionally, we present a modeling framework for conducting human cost-of-delay studies in obesity research. Future studies should incorporate the temporal dimension of intervention implementation to assess costs-of-delay and emphasize the urgency of timely action.

## Supporting information

S1 TRIPOD Checklist **From Collins GS, Moons KGM, Dhiman P, et al. BMJ 2024;385:e078378.** https://doi.org/10.1136/bmj-2023-078378. This is an Open Access article distributed in accordance with the terms of the Creative Commons Attribution (CC BY 4.0) license, which permits others to distribute, remix, adapt, and build upon this work, for commercial use, provided the original work is properly cited.
(PDF)

S1 Appendix Additional information on the simulation model and assumptions. (PDF)
