# Supplementary material for: Estimating the effect on obesity of delaying tax-based interventions in Mexico: A modeling study
Source: PLoS Med. 2025 Oct 23;22(10):e1004769. doi: 10.1371/journal.pmed.1004769 (PMC12548904; doi:10.1371/journal.pmed.1004769)
Supplement: S1 Appendix — (PDF) [file pmed.1004769.s002.pdf]

S1 Appendix for "The human cost of delaying interventions to reduce obesity: a modeling study using taxes in Mexico"

Martha Carnalla[1], Francisco Reyes-Sánchez[1], Alexis Alonso-Bastida[1], Alan Reyes-García[1], Alessio Hernández-Rojas[1], C. Gabriela García[1], Isabel Junquera-Badilla[1], Ana Basto-Abreu[1], Boyd Swimburn[2], Juan Rivera[1], and Tonatiuh Barrientos-Gutiérrez, PhD *[1]

[1] *Centro de Investigación en Salud Poblacional, Instituto Nacional de Salud Pública, México.*

[2] *School of Population Health, University of Auckland. New Zealand*

*Contact information: Center for Population Health Research, National Institute of Public Health, Avenida Universidad 655, Santa María Ahuacatitlán, 62100 Cuernavaca, Morelos, México, [tbarrientos@insp.mx], (52)5554871015.

Contents

1 Data sources **3**
1.1 Antropometric data 3
1.2 All-cause mortality by year 3

2 Population **4**

3 Intervention scenarios **5**
3.1 Historical trend in energy intake under the status quo 5
3.1.1 Changes in energy intake from 2000 5
3.1.2 Projecting the changes in energy intake to 2040 9
3.1.3 Changes in energy intake from 2021 over time 10
3.1.4 Projections of energy intake from 2021 11
3.2 Trend in energy intake for the intervention scenarios 11

4 Microsimulation model **13**
4.1 Summary of Hall et al. equations 13

5 Mortality from all causes **14**
5.1 Mortality from all causes by BMI 15
5.2 Estimation of the hazard rate of mortality for the reference BMI category in the Mexican population 16
5.2.1 Example of estimation of hazard rate 17

6 Annual deaths **18**

7 Uncertainty intervals **18**

8 Results **20**

9 Assumptions **24**

References **25**

1 Data sources

1.1 Antropometric data

We used anthropometric and sociodemographic data of the Mexican adult population from the National Health and Nutrition Surveys (ENSANUTs) 2020, 2021, and 2022. Each ENSANUT is a cross-sectional, multistage, stratified and cluster-sampled survey, that is designed to quantify the frequency and distribution of health and nutrition conditions in the Mexican population, and is representative at the national, regional, and area of residence level. The design and methods of each survey were described elsewhere. Each survey research protocol was approved by the Ethics, Biosafety, and Research Committees of the National Institute of Public Health of Mexico, and informed consent was obtained from each individual. Table A presents the sample size of the adult population aged 20 years or older by ENSANUT after data processing. In this table, we included the sample size of prior ENSANUTs as a reference.

Table A: Sample size of adults aged 20 or older from the National Health and Nutrition Survey (ENSANUT) by year. The sample sizes were taken after excluding individuals without weight or height data (missing data), extreme values of height and BMI, and pregnant or lactating women.

**Year**	**Sample size (n)**
2000	41,525
2006	32,655
2012	37,940
2016	7,770
2018	16,119
2020	9,768
2021	8,734
2022	8,352

Weight and height were collected by trained personnel using consistent validated and standardized methods across ENSANUT waves. For each wave, body weight was measured using digital scales with a precision of 100 g with participants wearing light clothing, and height was measured using stadiometers with a precision of 2 mm.

1.2 All-cause mortality by year

We considered projections of the Mexican population (mid-year population,years: 2021 to 2040) from the National Population Council of Mexico (CONAPO). [1]

Mortality was estimated by age and year. Table 1 in the main paper shows an example of the estimation for people aged 40 years in 2021.

2 Population

We simulated data corresponding to the Mexican adult population aged 20 years old from the ENSANUTs 2020-2022. We assumed that these ENSANUTs represented the population of the year 2021 (the mid-point between 2020 and 2022). The survey weights were divided by the number of ENSANUTs (= 3). Thus, they expanded to N = 254,655,982/3 = 84,885,327. Then, we adjusted the survey weights to match the population reported by CONAPO for 2021 (N = 84,812,069). The survey weights were adjusted by age using a sample balancing method called 'raking'. Raking is a statistical method that adjusts the sampling weights so that the population total of the sample matches the known population (the population reported by CONAPO). Table B shows the population of adults aged 20 and 25 taken from the ENSANUT 2020-2022 before calibration and the values reported by CONAPO for 2021. As mentioned before, we could match the survey weights to the values from CONAPO using raking.

Table B: Population of adults aged 20-25 estimated from the ENSANUT 2020-2022 and the population reported by the National Population Council of Mexico (CONAPO) in 2021

Age	ENSANUT	CONAPO
20	2,472,669	2,279,970
21	2,280,928	2,272,050
22	2,271,942	2,247,190
23	2,102,337	2,219,579
24	2,093,008	2,204,033
25	2,271,553	2,200,310

Raking was performed using the "survey" package on R Statistical Software [2–4].

```
# ----------------------   Code in R ----------------------- #

# --- Set the sampling design in R (not adjusted weights)

Svy.design.20_22 <- svydesign(id = ~id, strata = ~est_var,
weights = ~sampling.weights.20_22,
PSU = ~code_upm, data = Adults.ENSANUT.20_22)
options(survey.lonely.psu = "adjust")
```

```
# --- Raking:

calibration.raking <- calibrate(
Design = Svy.design.2022,
formula = ~ age, # Auxiliary variable
population =  ~ CONAPO.pop.by.age, #Population by age from CONAPO
calfun = "raking")

Adults.ENSANUT.20_22$raking.weights <- weights(calibration.raking)
# ---------------------------------------------------------------
```

3 Intervention scenarios

We simulated outcomes under the following scenarios

1) **Status quo:** This scenario considers the historical trend of total energy intake without intervention to estimate changes in population weight over time (Section 3.1).

2) **Doubling the tax on SSB and NEDFs:** The intervention scenario considers a proportional caloric reduction by doubling the tax on SSB and NEDFs: -0.85% of the total energy intake (TEI)

3.1 Historical trend in energy intake under the status quo

Figure A shows the steps followed to estimate the trend in energy intake under the status quo. More details are described in the next subsections.

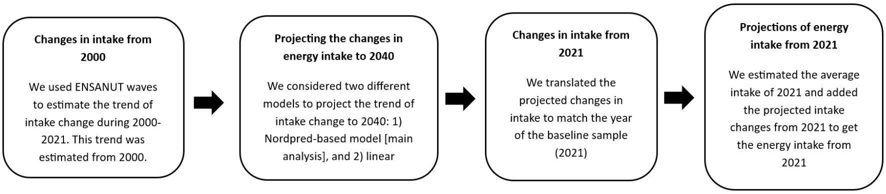

Fig A: Estimation of the trend of energy intake for the status quo scenario

3.1.1 Changes in energy intake from 2000

We estimated the Mexican energy intake trend for 2000-2021 considering changes from $T_0 = 2000$. Figure B shows the results of that estimation (the details are

presented below). We estimated that the energy intake of 2006 increased about 50 kcal/person with respect to 2000. The intake of 2021 compared to 2000 increased around 118 kcal/person.

Fig B: Changes in energy intake of the Mexican adult population from 2000, estimated using the National Health and Nutrition Surveys 2000, 2006, 2012, 2018, and 2020-2022

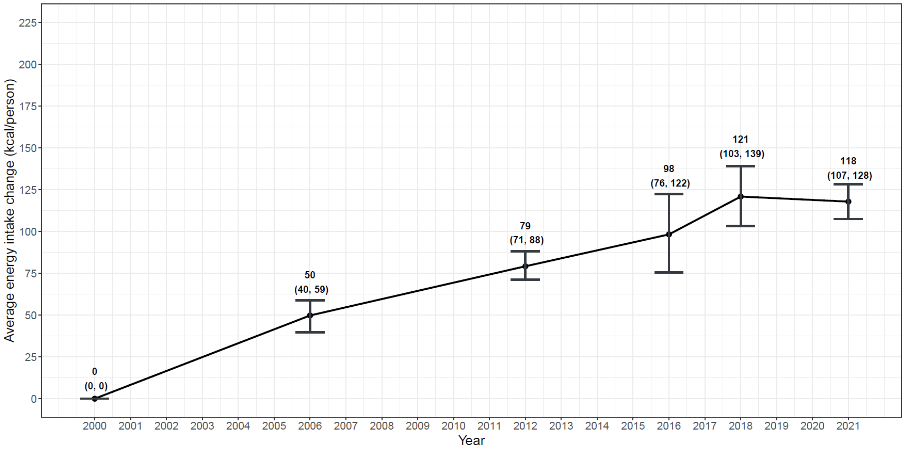

To estimate the values in Figure B, we used the following equation: [5]

$$\Delta I(T_n) \approx \frac{d\overline{BW}}{dt}(T_n) \times \phi_1 \text{ kcal/kg} + \left(\overline{BW}(T_n) - \overline{BW}(T_0)\right) \times \phi_2 \text{ kcal/kg/day}. \quad (1)$$

where $\Delta I(T_n)$ is the change in energy intake between T_0 and T_n (= ENSANUT 2000, 2006, 2012, 2016, 2018, 2020, or 2021-2022). $\overline{BW}(T_n)$ is the average body weight in the Mexican population at time T_n (estimated from the ENSANUT waves), and $d\overline{BW}/dt$ (T_n) is its rate of change at time T_n (kg/day). $\frac{d\overline{BW}}{dt}(T_n)$ was estimated assuming linear trends of body weight between ENSANUT waves:

$$\frac{d\overline{BW}}{dt}(T_n) = \frac{\overline{BW}(T_n) - \overline{BW}(T_{n-1})}{(T_n - T_{n-1}) \times 365 \text{ day/year}}; \quad (2)$$

where $n = 1, 2, 3,\ 4,\ 5,$ or 6, and $T_0 = 2000$. The coefficients ϕ_1 and ϕ_2 in equation (6) were estimated following Hall et al. 2009 methodology [5]:

$$\phi_1(\alpha_k) = \frac{1}{1-\beta} \times \left\{ \frac{\eta_F + \rho_F + \alpha_k \times \eta_{FFM} + \alpha_k \times \rho_{FFM}}{(1 + \alpha_k)} \right\}; \quad (3)$$

$$\phi_2(\alpha_k) = \frac{1}{1-\beta} \times \left\{ \frac{\gamma_F + \alpha_k \times \gamma_{FFM}}{1+\alpha_k} + \delta \right\}. \tag{4}$$

According to Hall, $\beta = 0.24$ considers the adaptation of energy expenditure during changes in diet; $\eta_F = 180$ kcal/kg and $\eta_{FFM} = 230$ kcal/kg account for the biochemical cost of tissue deposition, assuming that the change of fat mass (F) and fat-free mass (FFM) is primarily accounted by body protein and its associated water. The coefficients $\rho_F = 9400$ kcal/kg and $\rho_{FFM} = 1800$ kcal/kg are the energy densities for changes in F and FFM, respectively. $\gamma_F = 3.6$ kcal/kg/day and $\gamma_{FFM} = 22$ kcal/kg/day are regression coefficients explaining the relationship between the resting metabolic rate (dependant variable), and F and FFM, respectively [5,6]. Table C presents a summary of the parameters described.

Table C: Parameters used for estimating energy gaps, proposed by Hall et al.*

Parameter	Description	Value
β	Adaptive thermogenesis	0.24
δ	Physical activity coefficient	7.0
η_F	Cost of fat synthesis	180 kcal/kg
η_{FFM}	Cost of fat free tissue synthesis	230 kcal/kg
ρ_F	Energy density for changes in fat mass	9400 kcal/kg
ρ_{FFM}	Energy density for changes in fat-free mass	1800 kcal/kg
γ_F	Metabolic rate of adipose tissue	3.6 kcal/kg/day
γ_{FFM}	Metabolic rate of fat-free tissue	22.0 kcal/kg/day
C	Forbes body composition paramter	10.4

*Hall KD, Sacks G, Chandramohan D, Chow CC, Wang YC, Gortmaker SL, et al. Quantification of the effect of energy imbalance on bodyweight. The Lancet. 2011 8; 378(9793):826-37.

ϕ_1 and ϕ_2 in equations (3) and (4) depend only on α_k, which stands for the relative change in average F and FFM. α_k was estimated using Forbes' equation [7]:

$$\alpha_k := \frac{d(\overline{FFM_k})}{d\overline{F_k}} = \frac{C}{\overline{F}_{0k}} = \frac{10.4}{\overline{F}_{0k}}; \tag{5}$$

where $\overline{F}_0$ is the average fat mass at the intial time ($T_0 = 2000$). To estimate $\overline{F}_{0k}$, we first estimated fat mass for each individual in the ENSANUT 2000, using equations derived by Jackson et al. [8]:

$$F_j = \begin{cases} \left[(4.35 \times BMI_j)(0.05 \times BMI_j^2)46.24\right] \times BW_j & \text{for women} \\ \\ \left[(3.76 \times BMI_j)(0.04 \times BMI_j^2)47.80\right] \times BW_j & \text{for men} \end{cases}$$

where BMI_j and BW_j are the body mass index and the body weight of the individual j in the sample. Then, we estimated the average fat mass in 2000 ($\overline{F}_0$), considering the sampling design of the ENSANUT 2000. Table D shows the estimated values of $\overline{F}_0, \alpha$, ϕ_1, and ϕ_2. For the initial time T_0, we assumed that people's weight was in a steady-state, corresponding to a state of energy balance (energy intake = energy expenditure) [5]. In Hall's equation and microsimulation model, the energy balance assumption corresponds to no changes in energy intake at the initial time: $\overline{\Delta I}(T_0) \approx 0$

Table D: Parameters used to estimate changes in energy intake from 2000. $\overline{F}_0$ is the average fat mass in 2000, ϕ_1 (kcal/kg) and ϕ_2(kcal/kg/d) are parameters related to the rate of change in body weight and average body weight in the estimation of intake change, respectively.

**Parameter**	$\overline{F}_0$	ϕ_1	ϕ_2
Value	20.263	9235.837	22.15895

Using the values of the Table D, we can rewrite equation (6) as

$$\Delta I(T_n) \approx \frac{d\overline{BW}}{dt}(T_n) \times 9235.837 \text{ kcal/kg } + \left(\overline{BW}(T_n) - \overline{BW}(T_0)\right) \times 22.15895 \text{ kcal/kg/day.} \quad (6)$$

Table E presents the values of $\overline{BW}(T_n)$ by ENSANUT wave.

Table E: Average body weight estimated from the National Health and Nutrition Surveys (ENSANUTs) 2000, 2006, 2012, 2016, 2018, 2020-2022. The ENASNUT 2020-2022 represents the year 2021 (the midpoint betweeen 2020 and 2022).

	**Year**	$\overline{BW}$
T_0	2000	68.37
T_1	2006	70.26
T_2	2012	71.68
T_3	2016	72.56
T_4	2018	73.37
T_5	2021	73.60

3.1.2 Projecting the changes in energy intake to 2040

We projected the changes in energy intake up to 2040. We considered two different trends for the change in intake (Figure C): Nordpred-based projection (main), and a linear projection (sensitivity).

Fig C: Energy intake projections of the Mexican adult population. The solid gray line corresponds to a linear projection, and the black solid line corresponds to the Nordpred-based projection. The dashed lines corresponds to the uncertainty of the projections. The Nordpred-based fit starts with a linear trend (2000-2025), then gradual reductions are applied to its slope every 5 years. The linear slope of the Nordpred-based fit is reduced by 25% during 2026-2030, 50% during 2031-2035, and 75% for the subsequent years. The vertical line indicates the year in which the Nordpred-based projection begins to diverge from the linear projection (2026).

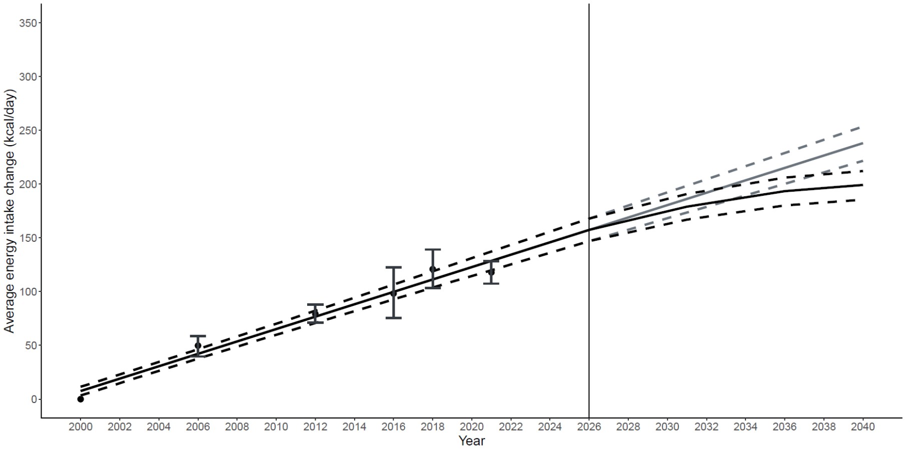

The linear model corresponds to the equation

$$\Delta I(T) = 5.762 \cdot year - 11517.101. \tag{7}$$

```
# ----------- Summary of the linear model in R -----------

Call:
lm(formula = DeltaI ~ year, data = .data)

Residuals:
      1        2        3        4        5        6
 -7.546    7.649    2.494   -1.503    9.601  -10.695
```

```
Coefficients:
             Estimate Std. Error t value Pr(>|t|)
(Intercept) -1.152e+04  1.034e+03  -11.14  0.00037 ***
year         5.762e+00  5.139e-01   11.21  0.00036 ***
---
Signif. codes:  0 '***' 0.001 '**' 0.01 '*' 0.05 ''. 0.1 ''  1

Residual standard error: 9.089 on 4 degrees of freedom
Multiple R-squared:  0.9692,    Adjusted R-squared:  0.9615
F-statistic: 125.7 on 1 and 4 DF,  p-value: 0.0003602

# ------------------------------------------------------------
```

3.1.3 Changes in energy intake from 2021 over time

We translated the changes in energy intake from T_0=2000 to $T_0^{'}$=2021 to match the year of the baseline sample:

$$\Delta I_{2021}(T) := \Delta I(T) - \Delta I(2021) \tag{8}$$

where $\Delta I_{2021}(T)$ is the projected intake change between $T_0^{'}$=2021 and time T (Figure D). With this translation, we obtain the intake changes from $T_0^{'} =$ 2021, needed to simulate with Hall's weight change model.

Fig D: Changes in energy intake of the Mexican adult population. The solid gray line corresponds to a linear projection, and the black solid line corresponds to the Nordpred-based projection. The dashed lines corresponds to the uncertainty of the projections.

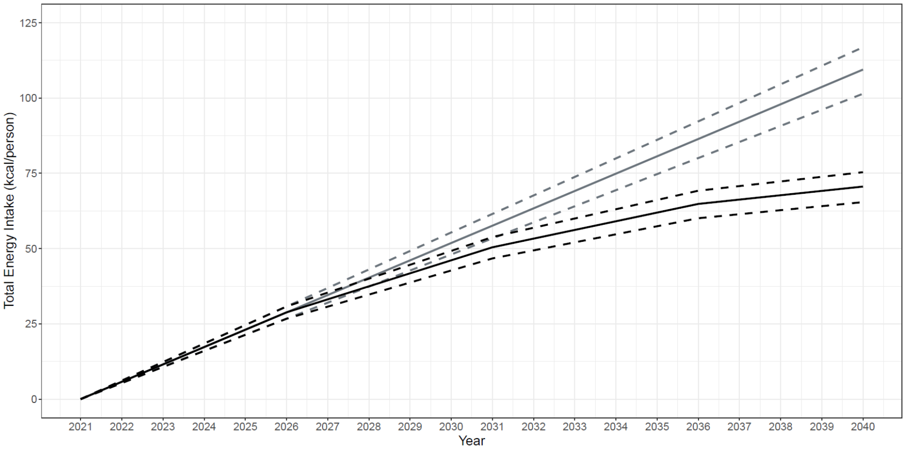

3.1.4 Projections of energy intake from 2021

For each individual in the baseline sample, we estimated the total energy intake using Mifflin et al. equation: [9]

$$TEI = \begin{cases} PAL \cdot \left[9.99 \cdot BW + 6.25 \cdot Height - 4.92 \cdot Age - 161\right] & \text{for women} \\ PAL \cdot \left[9.99 \cdot BW + 6.25 \cdot Height - 4.92 \cdot Age + 5\right] & \text{for men} \end{cases} \tag{9}$$

where BW, $Height$, and Age are the body weight (kg), height (cm), and age (years) of the individual. PAL denotes the physical activity level of the individual. We assumed $PAL = 1.5$ for all individuals, which corresponds to a sedentary level. Then, we estimated an average TEI in 2021 of 2165 kcal/person, and added the changes in intake from 2021 to get the projections of intake from 2021 (Figure E).

Fig E: Trends of the total energy intake. The solid gray line corresponds to a linear trend, and the solid black line corresponds to the Nordpred-based trend. The dashed lines corresponds to the uncertainty of the projections. Both the linear and Nordpred trends start at 2164 kcal (UI: 2157, 2172) in 2021. The linear trend presents a constant increase and, in 2040, ends at 2274 kcal (UI: 2261, 2287). Meanwhile, the Norpred trend ends at 2235 kcal (UI: 2225, 2246) and presents a non-linear behavior.

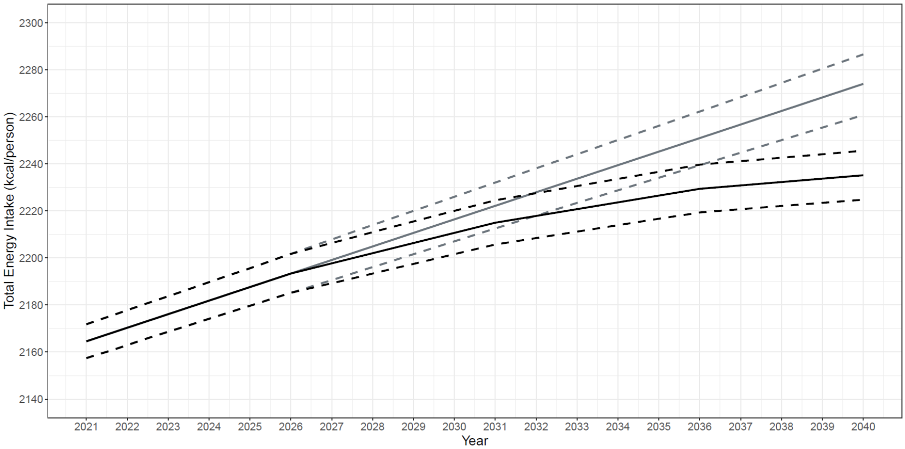

3.2 Trend in energy intake for the intervention scenarios

Figure F shows the energy intake trend for the intervention scenarios. The left graph presents the linear method according to the status quo (black line) and

tax scenarios (2025: dashed grey line "- -", 2030: dotted grey "..", and 2035: dot dashed grey line "-.-"). Due to linear behavior, the trends fluctuate constantly across the simulation. After two years, in the case of tax intervention, all the interventions converge in trajectory; therefore, at the end of the simulation, the caloric intake in the intervention scenarios is 2266 kcal. The right graph presents the scenarios under the Norpred trend; analog to linear cases in the Norpred trend, the caloric reduction converges through the simulation time until it finishes in 2216 kcal. When comparing the methods, Norpred has a reduced effect compared to the linear scenario, with a difference of 50 kcal at the end of the simulation.

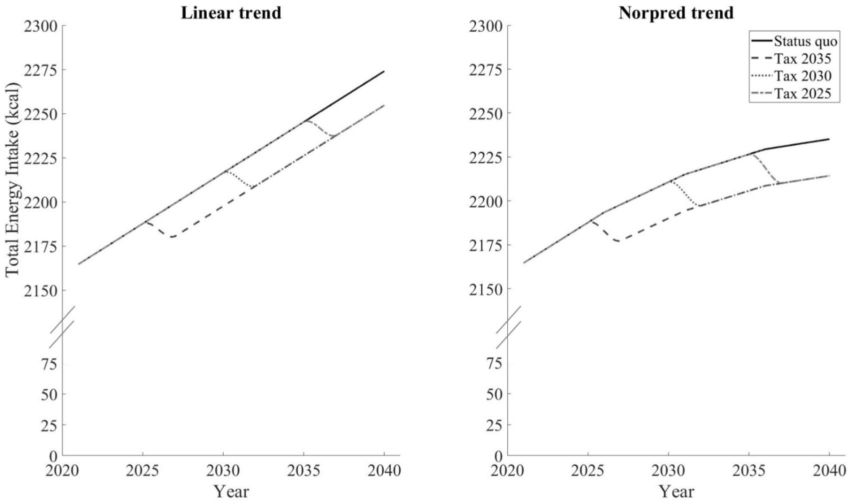

Fig F: Trends of the total energy intake.

Table F presents the results of total energy intake at the end of the simulation. We present the TEI according to the linear and Norpred trends. For each trend, the table presents the TEI for the Status quo and tax intervention (2025, 2030, and 2035). As mentioned, one of the principal characteristics of the different ways to use the intake trend is the final number of calories, which is less in the Norpred trend than in the linear trend.

Table F: Total energy intake at the end of simulation.

Scenario/Trend	Total Energy Intake (kcal; UI*)	
	**Linear**	**Norpred**
Status quo	2,274 (2,261 - 2,287)	2,235 (2225 - 2246)
Tax 2025	2,255 (2,238 - 2,266)	2,214 (2,202 - 2,246)
Tax 2030	2,255 (2,238 - 2,266)	2,214 (2,202 - 2,246)
Tax 2035	2,255 (2,238 - 2,266)	2,214 (2,202 - 2,246)

*UI = Uncertainty interval

4 Microsimulation model

4.1 Summary of Hall et al. equations

We simulated body weight using the microsimulation weight change model for adults proposed by Hall and colleagues [10]. To initialize the model, we used the individuals in the baseline sample considering their sex, age, weight, and height. The microsimulation model was completely programmed in the *bw* package [11] in *R*, and it was conducted using the projected changes in intake from 2021, detailed in the previous section. Body weight was simulated for each individual j in the sample as

$$BW_j(t) = BW_j^{\text{model}}\big(t + age_j;\ \text{Sex}_j, \text{Height}_j(0), \text{BW}_j(0), \overrightarrow{PAL}_j(t) = \overrightarrow{1.5}, \overrightarrow{\Delta Na}_j(t) = \overrightarrow{0}, EIchange_j\big) \tag{10}$$

where $\text{Height}_j(0)$ and $\text{BW}_j(0)$ are the initial height and body weight, respectively. $\overrightarrow{PAL_k}(t)$ is the physical activity level by day assumed to be constant over time (sedentary level = 1.5), and $\overrightarrow{\Delta Na_k}(t)$ the daily changes in sodium, accounting for changes in extracellular fluid. $EIchange_j$ represents the daily changes in energy intake from baseline for the individual j. In our case, the baseline is $T_0^{'} = 2021$ and

$$EIchange_j = \textit{Intake in 2021}_j \cdot \%\Delta I \tag{11}$$

where $\textit{Intake in 2021}_j$ is the total energy intake of the individual j in 2021 (estimated with the equation (9), and $\%\Delta I$ is percentage change in intake from baseline by day. $\%\Delta I$ was estimated as the projection of intake change from 2021 divided by the average intake in 2021 ($= \Delta I_{2021}(t)/I(0)$).

$$\%\Delta I = \left(\frac{\Delta I_{2021}(0)}{I(0)}, \frac{\Delta I_{2021}(1)}{I(0)}, ..., \frac{\Delta I_{2021}(u)}{I(0)}\right) \tag{12}$$

where the value "0" represents the baseline (2021), and $u = (2040-2021)/365$ is the last day of simulation. We used the relative change in intake ($\%\Delta I$) instead of the absolute values (ΔI) so that each individual had its own reduction in intake based on its initial intake.

5 Mortality from all causes

As mentioned in the main paper, we estimated the probability of all-cause mortality by age and year from national population proyections. Table G presents an example of the estimation process for adults aged 95 years in 2021. The expected deaths during the year 2021 for this group were estimated as the difference in total population between the years 2021 and 2022 (26,620 – 21,119 = 5,501). Then, the number of expected deaths during the year 2021 was divided by the population in 2021 to get the probability of all-cause mortality for this group in 2021 (5,501/26,620 = 0.0044). This procedure was repeated to estimate the annual probability of all-cause mortality up to the year = 2039 or age = 100, whichever occurs first. In the case of adults aged 95 years in 2021, they reach the age of 100 in 2026. When adults are 100 years old, We assumed a probability of all-cause mortality during the year of 100% (Table G).

Table G: Estimation of the annual probability of all-cause mortality for adults aged 95 in 2021 (mid-year) using the National Population Council of Mexico (CONAPO)

**Mid-year**	**Age**	**Population (CONAPO)**	**Expected deaths during the year all cause**	**Probability of all-cause mortality during the period** (year)
2021	95	26,620	5,501	20.7%
2022	96	21,119	4,121	19.5%
2023	97	16,998	3,536	20.8%
2024	98	13,462	2,980	22.1%
2025	99	10,482	2,466	23.5%
2026	100	8,016	8,016	100%[a]
2027	101	-	0	-
2028	102	-	0	-
2029	103	-	0	-
2030	104	-	0	-
...	...	-	0	-
2040	114	-	0	-

[a] Assumption: People aged 100 years had a probability of of 100% of all-cause mortality during the year.

Table H shows how we applied the probability of all-cause mortality for a

given individual aged 95 years in 2021 in the sample. The column "Probability of all-cause mortality (during the year)" corresponds to the values estimated in Table G. The age and survey weight of the individual were considered from the beginning of the year (period), starting from the baseline (2021). The survey weight of each individual in the sample was adjusted to consider the mortality estimated from CONAPO as

$$w_j(y) = \begin{cases} w_j(2021) & \textit{if } y = 2021(\text{baseline ENSANUT}) \\ w_j(y-1) \times \left[1 - PD_j(y-1)\right] & \textit{if } y > 2021 \end{cases}$$

where $w_j(y)$ is the survey weight of the individual j for the year y and $PD_j(y-1)$ is the probability of all-cause mortality during the year $y-1$.

Table H: Applying the probability of mortality to an individual aged 95 years in 2021. The survey weight of the year 2021 was taken from the ENSANUT 2020-2022. For the next years, the survey weight is adjusted to account for mortality. For example, for 2022, the survey weight was estimated as $10,467.0 \times (1 - 0.207) = 8,300.3$. Then, the survey weight for 2023 was $8,300.3 \times (1 - 0.195) = 6,681.8$. The same method was applied for years 2023-2026.

**Year**	**Probability of all-cause mortality during the period** (year)	**Age at the beginning of the period**	**Survey weight at the beginning of the period**
2021	20.7%	95	10,467.0 (initial)
2022	19.5%	96	8,300.3
2023	20.8%	97	6,681.8
2024	22.1%	98	5,292.0
2025	23.5%	99	4,122.4
2026	100%	100	0.0
2027	-	101	0.0

5.1 Mortality from all causes by BMI

From 2027, we estimated probabilities of all-cause mortality by BMI category for individuals aged 35 or more, using following equation: [12]

$$1 - P[D] = \sum_{i=1}^{6} \left\{ P[A_i] \times e^{-\lambda(HR_i)} \right\} \tag{13}$$

where $P[D]$ is the probability of all-cause mortality estimated from CONAPO (probability of all-cause mortality during the period). $P[A_i]$ and HR_i are the prevalence and the hazard ratio of the BMI category i, respectively. The HR_i used for the analyses are presented in Table 3 of the main paper. The equation (13) was solved to estimate λ (the hazard rate of mortality for the reference BMI category; $BMI \leq 25$). More details of the estimation of λ are presented in

the next subsection. Here, we present how equation (13) was derived following the methodology of Allison et, al. [12] First, the next equation is considered

$$1 - P[D] = \sum_{i=1}^{6} \{P[A_i] \times (1 - P[D|A_i])\} \tag{14}$$

$P[D|Ai]$ represents the proportion of deaths by all causes given the BMI category, which is what we want to estimate from that equation for each BMI category A_i. This equation expresses the proportion of survivals $(1 - P[D])$ decomposed as the sum of the proportion of survivals in each BMI category $(P[A_i] \times (1 - P[D|A_i]))$. To estimate the $P[D|A_i]$ from equation (14), we assumed that $1 - P[D|A_i]$ (survival probability in A_i) corresponded to an exponential survival distribution:

$$1 - P[D|A_i] = e^{-\lambda HR_i} \tag{15}$$

where λ is the mortality hazard rate for the BMI reference category (BMI $\leq$ 25 kg/m2), and HR_i is the mortality hazard ratio for the BMI category A_i (Table 1 in the main manuscript). In equation (15), $\lambda HR_i := \lambda_i$ approximates the hazard rate of the reference group A_i (for the reference group, $\lambda HR_i = \lambda \times 1 = \lambda$). Substituting equation (15) into equation (14), we get equation (13). Note that if we have λ, then we can estimate each $P[D|A_i]$ by substituting λ in equation 15. However, λ cannot be estimated analytically. We used a numerical method to estimate λ from equation 13.

5.2 Estimation of the hazard rate of mortality for the reference BMI category in the Mexican population

To determine the best value of λ in equation (13), we used the Gauss-Newton optimization method. This method is widely used in optimization problems, where the goal is to minimize the difference between the measured value and the value obtained concerning a system of equations. To do this, we need to define the parameters to be calculated in the system of equations, which will change within an iterative process until they meet a tolerance for the permissible error of the measurement to the defined system of equations. Please note that the focus of this work is not to explain the Gauss-Newton method in detail, but in Figure G, we present the pseudo-code of the optimization method used to find the optimal value of "λ". For more information on the demonstration of the optimization method, please refer to Qin et al. [13].

Fig G: Pseudo-code of the Gauss-Newton method.

Algorithm 1: Pseudo-code of the Gauss-Newton method.

Begin

Conditions of the Gauss-Newton algorithm

Tolerance (*tol*); Maximal number of iterations (ite_{max}); Initial conditions of the parameter to be characterized (λ_o), and the equation to be analyzed ($1 - \mathrm{P(D)}_o$).

Parameters of the equation

$$P(O) = 0.05 \quad h = 0.5$$
$$P(R) = 0.02 \quad q = 0.5$$

Development of the Jacobians concerning the parameter to be estimated in the equation.

$$\left(J = \frac{\partial \mathrm{P(O)e^{-h\lambda} + P(R)e^{-\lambda} + (1 - P(O) - P(R))e^{-q\lambda}}}{\partial\lambda}\right).$$

for (*i=1* to Number of probabilities of death analyzed)

for (*j=1* hasta ite_{max})

Parameter update

Evaluation of the probability of dying concerning the new parameter

$$\hat{x}_j = \partial \mathrm{P(O)e^{-h\hat{\lambda}} + P(R)e^{-\hat{\lambda}} + (1 - P(O) - P(R))e^{-q\hat{\lambda}}}$$

Evaluation of the Jacobians (J_k)

Calculation of the error (e_j) concerning the sample value and estimated value.

$$e_j = (1 - \mathrm{P(D)}_i) - (\hat{x}_j)$$

Gauss–Newton equation

$$\left(\hat{\lambda}_j = \hat{\lambda}_{j-1} - \left(J_j^T J_j\right)^{-1} J_j^T e_j\right)$$

if $\left(\left(J_j^T J_j\right)^{-1} J_j^T e\right) < tol$

Stop the parameter setting process.

$$j = ite_{max}$$

end if

end for (*j*)

end for (*i*)

End

5.2.1 Example of estimation of hazard rate

Let's suppose that we want to estimate the $P[D|Ai]$s for individuals aged 40 in the year 2021. Table I shows the $P[D]$ of 2021 (estimated as in Table G from CONAPO), and the distribution of the BMI categories ($P[Ai]$) for that group (estimated from the ENSANUT 2020-2022; baseline).

Table I: Probability of all-cause mortality and distribution of BMI categories of individuals aged 40 in 2021

Year	Probability of all-cause mortality during the year $P[D]$	BMI category						
		BMI<25.0 $P[A_1]$	25.0≤BMI<27.5 $P[A_2]$	27.5≤BMI<30.0 $P[A_3]$	30.0≤BMI<35.0 $P[A_4]$	35.0≤BMI<40.0 $P[A_5]$	40.0≤BMI $P[A_6]$	All BMI categories
2021	0.44%	19.6%	20.0%	19.8%	25.1%	12.4%	3.1%	100%

Considering the hazard ratios by BMI category shown in Table 1 in the main manuscript, the equation 13 can be written as:

$$1 - 0.0044 = \left\{0.196 \times e^{-\lambda}\right\} + \left\{0.20 \times e^{-\lambda(1.23)}\right\} + \left\{0.198 \times e^{-\lambda(1.52)}\right\} + \left\{0.251 \times e^{-\lambda(2.31)}\right\} + \left\{0.124 \times e^{-\lambda(3.51)}\right\} + \left\{0.031 \times e^{-\lambda(5.3)}\right\} \quad (16)$$

Using the numerical method, we estimated $\lambda = 0.002287207$. Then, $P[D|A1] = 1e{-}\lambda = 0.23\%$, $P[D|A2] = 1e^{-\lambda * 1.23} = 0.28\%$, $P[D|A3] = 0.35\%$, $P[D|A4] = 0.53\%$, $P[D|A5] = 0.8\%$, $P[D|A6] = 1.21\%$.

6 Annual deaths

In each scenario, annual deaths were estimated using the individuals' sampling weights and the probability of death of each individual ($PDj(y)$; Equation 1 in the main manuscript) as follows:

$$D(y) = \sum_{j=1}^{n} \left[PD_j(y) \times w_j(y)\right]$$

where $D(y)$ represents the number of deaths for a given year y, and n is the sample size of the baseline sample. $PDj(y)$ and $w_j(y)$ are the probability of death and the sampling weight of the individual j in the year y, respectively. For the next year $(y + 1)$, we updated the sampling weights to consider mortality:

$$w_j\,(y + 1) = \left[1 - PD_j(y)\right] \times w_j(y)$$

7 Uncertainty intervals

We accounted the uncertainty from the ENSANUT waves using the bootstrap method of Beaumont and Émond (2022), which is applicable to multistage stratified designs such as the ENSANUTs. [14]. This process was conducted in R using the package *svrep.* [15]

```
# ---------------------- Code in R -----------------------#

# ---- ENSANUT waves
```

```r
.year <- c(2000, 2006, 2012, 2016, 2018, 2021)

for(i in 1:length(.year)){

# ---- Set the design of the ENSANUT wave
Design <- svydesign(ids = ~id, strata= ~est_var,
                    weights=~svy_weights, PSU=~code_upm,
                    data= subset(ENSANUT_waves, year == .year[i]),
                    nest = TRUE)


# ---- Replicate 1000 survey weights
      boot_design <- as_bootstrap_design( Design,
                      type = 'Rao-Wu-Yue-Beaumont',
                      replicates = 1000 )


# ----- Get replicated weights
    assign( paste0( 'boot_weights', .year[i] ),
        weights(boot_design, type = 'analysis') )
}

# ---------------------------------------------------------
```

For each bootstrap subsample, we re-estimated the projection of intake simulating the parameters in Table C, indicated as "**Monte Carlo**" in Table J.

Table J: Parameters and equations included in the model

Parameter	Description	Value (95% CI)	Data source	Accounted uncertainty in the model	Caveats
Weight	Measured body weight (kg)	Average: 73.6 (73.3, 73.9)	National health and Nutrition Survey 2020-2022	**Sampling design** Accounted using Bootstrap	
Height	Measured height (meters)	Average: 1.60 (1.59, 1.60)	National health and Nutrition Survey 2020-2022	**Sampling design** Accounted using Bootstrap	
Body fat percentage (%BF)	%	%BF (men) = 3.76 $\times BMI -$ 0.04 $\times BMI^2 - 47.80$; %BF (women) = 4.35 $\times BMI -$ 0.05 $\times BMI^2 - 46.24$	Jackson et al, 2002	**Sampling design** Accounted using Bootstrap	
Resting Energy Expenditure (REE)	Kcal	REE (men) = 10*weight (kg) + 6.25*height (cm) – 5*age (years) + 5; REE (women) = 10*weight (kg) + 6.25*height (cm) – 5*age (years) - 161	Mifflin et al, 1990.	**Sampling design** Accounted using Bootstrap	
Doubling SBB and NEDF tax	Expected change in consumption of industrialized beverages and foods due to doubling tax	-7.6% (NA[b]) from beverages -6.0% (-8.2, -3.8) of from foods	Colchero et al., 2017 Taillie et al., 2016	**Monte Carlo** The parameter value was randomly selected assuming a normal distribution, using its mean and standard deviation	Reduction in purchases will be completely translated in consumption.
Trend of energy intake	Kcal increase per year in TEI		Recalibrated from Reyes-Sánchez et al., 2023	**Monte Carlo** The parameters shown in Table C were randomly selected assuming a normal distribution, using their mean and standard deviation. + **Sampling design** Using Bootstrap to account for uncertainty of average body weights from the ENSANUTs	The trend considers that population behavior is the same without externalities or other interventions.
All-cause mortality risk by BMI	Hazard ratios for all-cause mortality by BMI category and age-group	Reference group in all group ages: BMI $\leq$ 25 kg/m2 *35-49 years* HR per 5 unit of BMI 1.52 (1.47, 1.56) *50-69 years* HR per 5 unit of BMI 1.37 (1.35, 1.39) *70-89 years* HR per 5 unit of BMI 1.21 (1.17, 1.25)	Global BMI Mortality Collaboration, 2016	**Monte Carlo** The parameters were randomly selected assuming a normal distribution, using their corresponding mean and standard deviation	We are considering no differential mortality risk by BMI in people aged 20 to 34 years, and we are considering the same HR in individuals 90 to 100 years as the HR of the 70–89-year-olds.

[a] sd = standard deviation
[b] NA = Not Available

8 Results

Figure H presents the results of BMI evolution across the simulation. The left graph shows the BMI behavior of the Status quo and tax interventions concerning the linear trend intake, while the right graph presents the BMI behavior with Norpred trend intake. Comparing the two graphs, the BMI reaches higher values of BMI in the linear trend. The Norpred scenario integrates a dynamic trend to stabilize from the 2035-year simulation; this effect gives the

principal characteristic of Norpred in contrast to the Linear trend.

Fig H: Evolution of BMI in a closed cohort (18-100 years) according to linear and Norpred trends since 2021.

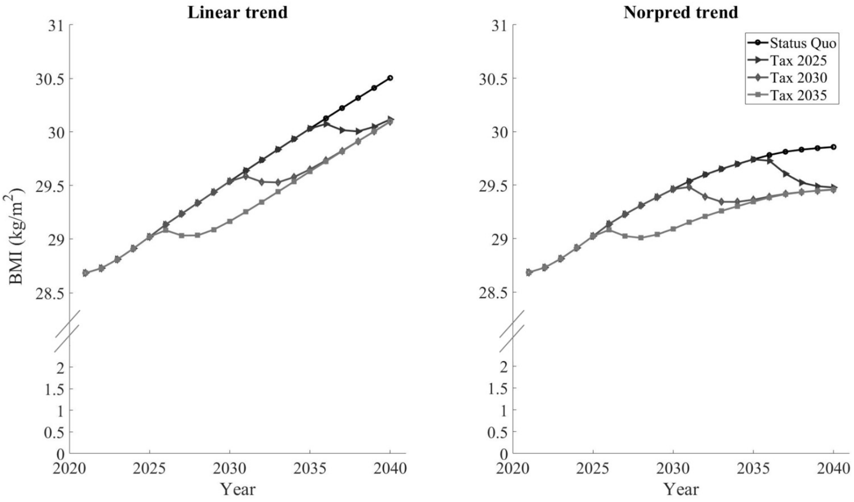

Figure I describes the evolution of Obesity prevalence from 2021 to 2040. Analog to BMI changes, obesity prevalences have a similar behavior regarding total energy intake, in the same way as linear and Norpred trends. In the case of linear trends, the Status quo scenario reaches a value of 49.3% (UI: 47.8, 50.8) of obesity prevalence in 2040, and with an intervention, the obesity prevalence converges to 46.3% (UI: 44.4, 48.1). In Norpred scenarios, there exists a significant difference between linear cases; for the Status quo scenario, the obesity prevalence ends at 44.5% (UI: 43.1, 45.8), and for the tax cases, it converges at 41.6% (UI:40.2, 43.1) at the end of the simulation.

Fig I: Evolution of Obesity prevalence in a closed cohort (20-100 years) according to Linear and Norpred trends since 2021

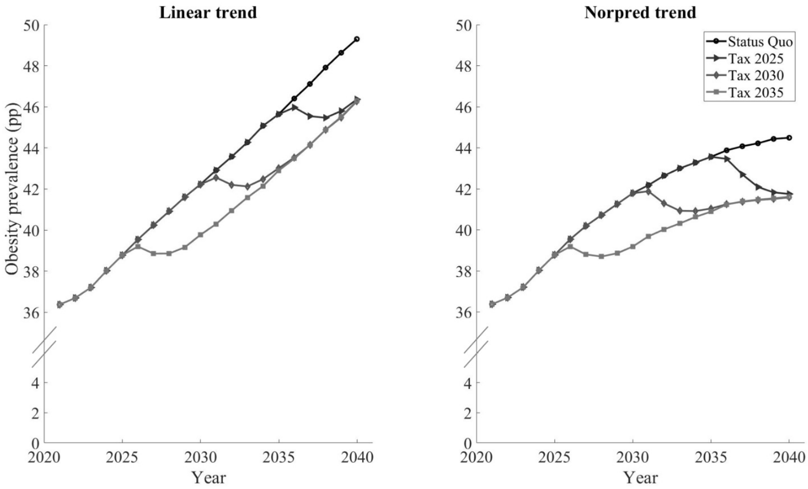

Table K presents the main results of the scenarios regarding intake trends from 2021 to 2040. This table contains the last estimation in 2040 of the evolution of BMI, and Obesity prevalence through the tax intervention. One of the principal differences between the scenarios with the linear trend and Norpred trend is the magnitude of results in almost all the variables analyzed, except for the variable of deaths when the difference is null due to CONAPO projections used in the Status quo scenario as reference. In obesity prevalence, the linear case is approximately 10% bigger than the Norpred scenario.

Table K: Main results of the proposed scenarios at the end of the simulation.

**Scenario/Trend**	**BMI** (kg/m^2)		**Obesity prevalence (pp)**	
	**Linear**	**Norpred**	**Linear**	**Norpred**
Status quo	30.5 (30.3, 30.7)	29.9 (29.7, 30)	49.3 (47.8, 50.8)	44.5 (43.2, 45.8)
Tax 2025	30.1 (29.9, 30.3)	30.1 (29.9, 30.3)	46.3 (44.4, 48.1)	41.6 (40.2, 43.1)
Tax 2030	30.1 (29.8, 30.3)	29.5 (29.3, 29.7)	46.3 (44.4, 48.1)	41.6 (40.2, 43)
Tax 2035	30.1 (29.9, 30.4)	29.5 (29.3, 29.7)	46.3 (44.4, 48.1)	41.7 (40.4, 43.1)

Table L presents the obesity prevalence (%) by year for the status quo scenario.

Table L: Projected obesity prevalence for the Mexican adult population from 2021 to 2040

Year	Nordpred-based model				Linear model			
	Status quo	Tax in 2035	Tax in 2030	Tax in 2025	Status quo	Tax in 2035	Tax in 2030	Tax in 2025
2021	36.4 (35.6, 37.1)	36.4 (35.6, 37.1)	36.4 (35.6, 37.1)	36.4 (35.6, 37.1)	36.4 (35.6, 37.1)	36.4 (35.6, 37.1)	36.4 (35.6, 37.1)	36.4 (35.6, 37.1)
2022	36.7 (35.9, 37.5)	36.7 (35.9, 37.5)	36.7 (35.9, 37.5)	36.7 (35.9, 37.5)	36.7 (35.9, 37.5)	36.7 (35.9, 37.5)	36.7 (35.9, 37.5)	36.7 (35.9, 37.5)
2023	37.2 (36.4, 38)	37.2 (36.4, 38)	37.2 (36.4, 38)	37.2 (36.4, 38)	37.2 (36.4, 38)	37.2 (36.4, 38)	37.2 (36.4, 38)	37.2 (36.4, 38)
2024	38 (37.2, 38.8)	38 (37.2, 38.8)	38 (37.2, 38.8)	38 (37.2, 38.8)	38 (37.2, 38.8)	38 (37.2, 38.8)	38 (37.2, 38.8)	38 (37.2, 38.8)
2025	38.8 (37.9, 39.7)	38.8 (37.9, 39.7)	38.8 (37.9, 39.7)	38.8 (37.9, 39.7)	38.8 (37.9, 39.7)	38.8 (37.9, 39.7)	38.8 (37.9, 39.7)	38.8 (37.9, 39.7)
2026	39.5 (38.5, 40.5)	39.5 (38.5, 40.5)	39.5 (38.5, 40.5)	39.2 (38.2, 40.2)	39.5 (38.5, 40.5)	39.5 (38.5, 40.5)	39.5 (38.5, 40.5)	39.2 (38.2, 40.2)
2027	40.2 (39.2, 41.2)	40.2 (39.2, 41.2)	40.2 (39.2, 41.2)	38.8 (37.8, 39.8)	40.2 (39.3, 41.1)	40.2 (39.2, 41.2)	40.2 (39.3, 41.1)	38.9 (38, 39.8)
2028	40.7 (39.7, 41.7)	40.7 (39.6, 41.9)	40.7 (39.7, 41.7)	38.7 (37.7, 39.7)	40.9 (39.9, 41.9)	40.9 (39.8, 42)	40.9 (39.9, 41.9)	38.9 (37.9, 39.9)
2029	41.3 (40.2, 42.3)	41.3 (40.1, 42.5)	41.3 (40.2, 42.3)	38.9 (37.9, 39.9)	41.6 (40.5, 42.7)	41.6 (40.4, 42.8)	41.6 (40.5, 42.7)	39.2 (38.1, 40.3)
2030	41.8 (40.8, 42.8)	41.8 (40.6, 43)	41.8 (40.8, 42.8)	39.2 (38.2, 40.2)	42.2 (41.1, 43.3)	42.2 (41, 43.5)	42.2 (41.1, 43.3)	39.8 (38.7, 40.9)
2031	42.2 (41.2, 43.2)	42.2 (41, 43.5)	41.9 (40.8, 43)	39.7 (38.7, 40.8)	42.9 (41.8, 44)	42.9 (41.6, 44.2)	42.6 (41.5, 43.8)	40.3 (39.1, 41.4)
2032	42.6 (41.5, 43.7)	42.6 (41.3, 43.9)	41.3 (40.1, 42.4)	40 (38.9, 41.1)	43.6 (42.5, 44.8)	43.6 (42.2, 45)	42.2 (41, 43.4)	40.9 (39.8, 42)
2033	43 (41.8, 44.2)	43 (41.7, 44.3)	40.9 (39.7, 42.1)	40.3 (39.1, 41.5)	44.3 (43.1, 45.5)	44.3 (43, 45.6)	42.1 (40.9, 43.4)	41.6 (40.4, 42.8)
2034	43.3 (42.1, 44.4)	43.3 (41.9, 44.6)	40.9 (39.6, 42.2)	40.6 (39.5, 41.8)	45.1 (43.8, 46.4)	45.1 (43.7, 46.5)	42.5 (41.2, 43.8)	42.1 (40.8, 43.4)
2035	43.6 (42.4, 44.8)	43.6 (42.2, 45)	41 (39.7, 42.3)	40.9 (39.7, 42.1)	45.6 (44.2, 47)	45.6 (44.2, 47)	43 (41.5, 44.5)	42.9 (41.5, 44.2)
2036	43.9 (42.7, 45.1)	43.4 (42, 44.8)	41.2 (39.9, 42.5)	41.2 (40, 42.5)	46.4 (45, 47.8)	45.9 (44.4, 47.4)	43.5 (42, 45)	43.5 (42, 45)
2037	44.1 (42.9, 45.4)	42.6 (41.2, 44)	41.4 (40, 42.8)	41.4 (40.1, 42.6)	47.1 (45.6, 48.6)	45.5 (44, 47)	44.1 (42.5, 45.7)	44.2 (42.7, 45.7)
2038	44.2 (42.9, 45.5)	42 (40.6, 43.4)	41.5 (40.1, 42.9)	41.5 (40.2, 42.8)	47.9 (46.4, 49.4)	45.4 (43.8, 47)	44.9 (43.3, 46.5)	44.9 (43.3, 46.5)
2039	44.4 (43, 45.8)	41.8 (40.4, 43.2)	41.5 (40.1, 42.9)	41.5 (40.1, 42.9)	48.6 (47, 50.2)	45.8 (44.1, 47.5)	45.5 (43.8, 47.2)	45.5 (43.8, 47.2)
2040	44.5 (43.2, 45.8)	41.7 (40.2, 43.2)	41.6 (40.2, 43)	41.6 (40.2, 43)	49.3 (47.8, 50.8)	46.3 (44.4, 48.1)	46.3 (44.4, 48.1)	46.3 (44.4, 48.1)

Table M shows the accumulated averted deaths and the years lived without obesity estimated with the linear projection of energy intake.

Table M: Predicted cumulative deaths averted and years lived without obesity (YLWO) from 2021 to 2040 due to doubling the SSB and NEDF tax in years 2025, 2030 and 2040. Population: Mexican adults aged 20 and over. Model: Linear Model of Energy Intake.

**Scenario**	**Deaths averted (thousands)** (p2.5 – p97.5)	**YLWO saved (thousands)** (p2.5 – p97.5)
Doubling tax in 2025	173.6 (134.7 – 212.5)	25,629.6 (19,496.0 – 31,763.2)
Doubling tax in 2030	113.7 (88.3 – 139.1)	14,877.1 (11,324.6 – 18,429.6)
Doubling tax in 2035	42.5 (32.4 – 52.6)	4,906 (3,749.2 – 6062.7)

9 Assumptions

1. The projection of TEI under status quo has three assumptions: (1) no dietary changes (e.g., stable processed food consumption), (2) no policy/market shifts affecting food availability or preferences, and (3) fixed consumption habits independent of secular trends (e.g., urbanization effects) and were previously validated empirically through agreement with linear, root square, and Gompertz fits in Mexican population data [16], and are consistent externally with multinomial obesity projections in the US [17].

2. We assumed that the reduction of TEI (-9.5%) would be sustained over time for the tax scenario with no attenuation over time because since 2009 the tax has been adjusted by inflation. For example, if the average TEI in the status quo of the years 2028 and 2037 are 2200 kcal/day and 2350 kcal/day, respectively, the average reductions of intake would be 21 kcal/day (= 2200*0.0095) in 2028 and 22 kcal/day (=2350*0.0095) in 2037. We did not consider a trend in SSB or NEDF consumption. The tax is the only influence on the energy intake trend, with no other external influence (i.e., other interventions implemented during the modeling time).

3. Mortality could increase depending on the years lived with obesity of the individuals (years of exposure to the risk factor). For example, individuals who have lived one year with obesity have a lower probability of dying than individuals who have lived five years with obesity. We are not accounting for this potential increase in mortality across individuals with different years living with obesity [18].

References

[1] Consejo Nacional de Poblacion. Proyecciones de la población en México y de las Entidades Federativas, 2020–2070. Available from: https://datos.gob.mx/busca/dataset/proyecciones-de-la-poblacion-de-mexico-y-de-las-entidades-federativas-2020-2070.

[2] Lumley T. Complex Surveys: A Guide to Analysis Using R: A Guide to Analysis Using R. John Wiley and Sons; 2010.

[3] Lumley T. survey: analysis of complex survey samples; 2020.

[4] R Core Team. R: A Language and Environment for Statistical Computing. Vienna, Austria: R Foundation for Statistical Computing; 2021. Available from: https://www.R-project.org/.

[5] Hall KD, Guo J, Dore M, Chow CC. The Progressive Increase of Food Waste in America and Its Environmental Impact. PLOS ONE. 2009 11;4(11):e7940. Available from: https://doi.org/10.1371/journal.pone.0007940.

[6] Nelson KM, Weinsier RL, Long CL, Schutz Y. Prediction of resting energy expenditure from fat-free mass and fat mass. The American Journal of Clinical Nutrition. 1992 11;56(5):848-56.

[7] Forbes GB. Lean Body Mass-Body Fat Interrelationships in Humans. Nutrition Reviews. 2009 4;45(10):225-31.

[8] Jackson A, Stanforth P, Gagnon J, Rankinen T, Leon A, Rao D, et al. The effect of sex, age and race on estimating percentage body fat from body mass index: The Heritage Family Study. International Journal of Obesity. 2002 6;26(6):789-96.

[9] Mifflin M, St Jeor S, Hill L, Scott B, Daugherty S, Koh Y. A new predictive equation for resting energy expenditure in healthy individuals. The American Journal of Clinical Nutrition. 1990 2;51(2):241-7.

[10] Hall KD, Sacks G, Chandramohan D, Chow CC, Wang YC, Gortmaker SL, et al. Quantification of the effect of energy imbalance on bodyweight. The Lancet. 2011 8;378(9793):826-37.

[11] Camacho-García-Formentí D, Zepeda-Tello R. bw; 2018.

[12] Allison DB, Fontaine KR, Manson JE, Stevens J, VanItallie TB. Annual deaths attributable to obesity in the United States. Jama. 1999;282(16):1530-8.

[13] Qin Y, Kavetski D, Kuczera G. A robust Gauss-Newton algorithm for the optimization of hydrological models: From standard Gauss-Newton to robust Gauss-Newton. Water Resources Research. 2018;54(11):9655-83.

[14] Beaumont JF, Émond N. A Bootstrap Variance Estimation Method for Multistage Sampling and Two-Phase Sampling When Poisson Sampling Is Used at the Second Phase. Stats. 2022 3;5(2):339-57.

[15] Schneider B. svrep: Tools for Creating, Updating, and Analyzing Survey Replicate Weights; 2023. R package version 0.6.0. Available from: https://CRAN.R-project.org/package=svrep.

[16] Reyes-Sánchez F, Basto-Abreu A, Torres-Álvarez R, Carnalla-Cortés M, Reyes-García A, Swinburn B, et al. Caloric reductions needed to achieve obesity goals in Mexico for 2030 and 2040: A modeling study. PLOS Medicine. 2023 06;20(6):1-16. Available from: https://doi.org/10.1371/journal.pmed.1004248.

[17] Ward ZJ, Bleich SN, Cradock AL, Barrett JL, Giles CM, Flax C, et al. Projected U.S. State-Level Prevalence of Adult Obesity and Severe Obesity. New England Journal of Medicine. 2019;381(25):2440-50. Available from: https://www.nejm.org/doi/full/10.1056/NEJMsa1909301.

[18] Abdullah A, Wolfe R, Stoelwinder JU, de Courten M, Stevenson C, Walls HL, et al. The number of years lived with obesity and the risk of all-cause and cause-specific mortality. International Journal of Epidemiology. 2011 02;40(4):985-96. Available from: https://doi.org/10.1093/ije/dyr018.
